# Assessment of root morphology and canal configuration of maxillary premolars in a Saudi subpopulation: a cone-beam computed tomographic study

**DOI:** 10.1186/s12903-021-01739-1

**Published:** 2021-08-13

**Authors:** Saad M. Al‑Zubaidi, Moazzy I. Almansour, Nada N. Al Mansour, Ahad S. Alshammari, Ahad F. Alshammari, Yazeed S. Altamimi, Ahmed A. Madfa

**Affiliations:** 1 Department of Restorative Dental Science, Collage of Dentistry, University of Ha’il, Ha’il, Kingdom of Saudi Arabia; 2 Ministry of Health, Ha’il, Kingdom of Saudi Arabia

**Keywords:** Cone beam computed tomography, Canal morphology, Maxillary premolars, Saudi subpopulation

## Abstract

**Background:**

The objective of this study was to use CBCT to look into the root canal morphology of maxillary premolars in a Saudi Arabian subpopulation and associate the results to existing canal morphology classifications.

**Methods:**

The sample size for this analysis was 500 right and left untreated maxillary first and second premolars with fully formed roots from 250 Saudi residents (125 male and 125 female). The following observations were made using CBCT on the teeth related: (1) The number and morphology of roots; (2) The canal morphology for each root according to Vertucci's classification. The frequency and similarities between the right and left sides, as well as between females and males, were studied. The Chi-square test was used to assess the results.

**Results:**

Of the 500 maxillary first premolars studied, 199 teeth had one root (39.8%), whilst 293 (58.6%) were two-rooted. Three-rooted maxillary first premolars were found in 8 (1.6%). For maxillary second premolars, 416 premolars had one root (83.2%), whilst 79 (15.8%) had two roots and the rest 5 (1.0%) were three roots. There were significant differences of number of root were found between groups (p > 0.05). For maxillary first premolar, Type IV was the most frequent, accounting for 57.8% of the sample (n = 289), followed by type II (32.8%, n = 164). For maxillary second premolar, Type I was mainly occurrence 302 (60.4%), followed by Type II (16.4%, n = 82).

**Conclusions:**

The majority of maxillary first premolars had two roots and two root canals, while one root and one root canal was the most common anatomical configuration for maxillary second premolars. Additional canal forms do occur on occasion, and clinicians should pay close attention to them.

## Background

The science of root canal care is founded on the anatomy of the base tooth. Today, root apex is not the only field of endodontic research, but the concept of three-dimensional root canal filling implies that, while working length and maintaining it are more important, access to all canal within complications is also essential to facilitate root canal filling [1].

Failure to consider differences in root and canal morphologies is the most common cause of failed root canal care. To avoid endodontic failure due to incomplete debridement and obturation, a detailed knowledge of the anatomy of the teeth and an expectation of their possible deviations is essential [1]. Previous research has found that different populations have different patterns in the number and shape of roots and canals [2–6], which tend to be hereditarily determined [7–9] and are significant for locating population ethnic backgrounds. As a result, it is critical to be aware with the differences in tooth morphology and distinguishing structures among different ethnic groups, as this information can help with canal position and negotiation, as well as their subsequent management [10].

Current research has shown that the root canal system is not a single canal that runs uniformly from orifice to apex, but rather a complex system that splits and joins canals along the way to the apex [11–13]. A root canal begins with an orifice in the pulp chamber and ends with an apical foramen in the periodontium. Root canals offer a number of configurations from tooth to tooth in different as well as the same individual during their course [14].

The maxillary premolars are considered among the most problematic teeth during endodontic treatment because of various of the root canal configuration [15]. Furthermore, the root canal morphology of maxillary premolars has been found to be highly variable [16–19]. Clinical treatment of maxillary premolars with unpredictably morphological roots and canals can be difficult [20, 21]. Among the difficulties are repeated endodontic treatment errors due to missing canals or the inability to radiographically image the apical limit of multi-rooted premolars. As a result, a detailed information of the anatomical features of the root canal system in the maxillary premolars is critical for improving root canal therapy and post core restoration success rates while also reducing complications.

Root modeling, sectioning, tooth-clearing procedure, radiographic inspection, cone-beam computed tomography (CBCT), and micro-computed tomography (micro-CT) imaging are some of the methods used to assess the anatomy and morphology of root canals [22–27]. Neelakantan et al. [28] compared the effectiveness of four tomography methods with tooth-clearing technique and a digital radiography. They found that peripheral quantitative computed tomography and CBCT were as effective as tooth-clearing technique and canal staining in recognizing root canal systems. While micro-CT has grown in popularity as a result of its precision, high resolution, and ability to perform comprehensive qualitative and quantitative measurements of root canal anatomy, it is not available in every country. In addition, the cost and radiation dose of micro-CT are important considerations.

The objective of this research was to use CBCT to look into the root canal morphology of maxillary premolars in a Saudi Arabian subpopulation and associate the results to existing canal morphology classifications.

## Method

Five hundred individuals (250 females and 250 males) were registered in this study, from those who attending the radiologic diagnostic center for three-dimensional radiological scanning in the period between May 2017 and November 2019. Informed consent was waived by the ethics committee of college of Dentistry, University of Hail due to retrospective nature of the study. The patients that taking CBCT scanning for diagnostic purposes of maxillary premolars were collected. The records were reviewed retrospectively. All of the reports analyzed belonged to patients who had been treated at the Hail clinics. Any photographs, radiographs, or test results collected during care can be used for academic and research purposes, but no personally identifiable details will be disclosed.

A database of 3000 CBCT scans was analyzed, and 500 of them met the study's inclusion criteria: non-distorted CBCT scans of maxillary premolars with completely developed roots in patients aged 18 to 60 years. Images of teeth treated endodontically or with postcoronal restorations, metallic restorations, full-coverage restorations, or those causing scan artifacts were removed. Teeth with root resorption or calcification or teeth associated with periapical lesions and low-quality CBCT images were also excluded. Anatomical symmetry was determined by comparing scans that involved teeth on both sides. The final sample size in this study was 1000 CBCT images after examination of the 3000 images according to the inclusion/exclusion criteria.

The CBCT machine used for the scans was the Carestream CS 8100 3D (Carestream Dent LLC, Atlanta, USA). X-ray generator specified with 60–90 kV, 2–15 mA and 140 kHz. This machine had the following parameters: a CMOS sensor with Dental Volumetric Reconstruction (DVR), scan time of 3 to 15 s, fields of view (FOV) are 4 × 4, 5 × 5, 8 × 5 and 8 × 8 cm, and voxel size 75 µm minimum. Analyzing the images was performed using the CS 3D Imaging Software (Carestream Dent LLC, Atlanta, USA).

Axial, sagittal, and coronal two-dimensional sections of each scan were displayed and data were recorded. Image contrast and brightness were changed as needed using the image processing function in the utilized program to achieve best display and visualization.

The following observations were made using CBCT on the teeth related:

- The number and morphology of roots
- The canal morphology for each root according to Vertucci's classification.

Before evaluation, all examiners were participated in a calibration training. All of the CBCT images were assessed by endodontists with at least 5 years’ experience. To ensure the validity of the study's findings, 30 CBCT images were drawn at random to assess inter-examiner reliability by recording root canal numbers and determining the type of root canal system configuration based on Vertucci's classification. The inter-examiner reliability and intra-examiner reliability were analyzed.

The statistical package for the social sciences, version 22.0, was used to examine the results (SPSS Inc., Chicago, IL, USA). The total number of roots, root canal configuration, and unilateral and bilateral occurrences were all investigated. The frequency and similarities between the right and left sides, as well as between females and males, were studied. The Chi-square test was used to assess the results. Statistical significance was identified at the level of *P* < 0.05.

## Results

The analysis of each tooth was done independently by investigators following calibration of the researchers with the supervisors based on the anatomical criteria and variations utilized in this study, and was repeated after a two-week interval. The observers' readings were compared, and if there was disagreement in the analysis and interpretation of the radiographic data, a consensus was established following a conversation among the four students. The Kappa value for the intra-observer agreement was 0.93 for both observers and 0.89 for the inter-observer agreement.

Table 1 summarizes the number of roots in relation to gender and tooth position on right and left sides. Of the 1000 maxillary premolars assessed, 615 teeth had single-rooted (61.5%), while 177 (35.4%) were two-rooted. Three-rooted maxillary premolars were found in 13 (1.3%). Three-rooted maxillary premolars were found totally in female 13 (1.3%). For gender, there were statistically significant difference between groups (*p* < 0.05). However, for tooth position, no significant differences of number of root were found between groups (*p* > 0.05).

**Table 1:** Number of roots for gender and tooth position

Number of roots	Gender			Tooth position		
	Male	Female	Total	Left side	Right side	Total
One root *n* (%)	247 (53.0)	368 (68.9)	615 (61.5)	316 (63.2)	299 (59.8)	615 (61.5)
Two roots *n* (%)	206 (44.2)	166 (31.1)	372 (37.2)	177 (35.4)	195 (39.0)	372 (37.2)
Three roots *n* (%)	13 (2.8)	0 (0)	13 (1.3)	7 (1.4)	6 (1.2)	13 (1.3)

Chi-square, Fisher’s Exact tests; for gender *p* < 0.05; for side *p* > 0.05

For the number of root canal offices, 387 premolars had one canal orifice (38.7%), whilst 600 (60.0%) had two canal orifices and the rest 13 (1.3%) were three root canal orifices as shown in Table 2. The three root canal orifices were totally presented in females 13 (1.3%). A significant difference of number of root canal orifices were found between females and males (*p* < 0.05). However, there were no significantly different were found between left and right (*p* > 0.05).

**Table 2:** Number of canal office for gender and tooth position

Number of roots	Gender			Tooth position		
	Male	Female	Total	Left side	Right side	Total
One-orifice *n* (%)	160 (34.3)	227 (42.5)	387 (38.7)	193 (38.6)	194 (38.8)	387 (38.7)
Two-orifice *n* (%)	293 (62.9)	307 (57.5)	600 (60.0)	300 (60.0)	300 (60.0)	600 (60.0)
Three-orifice *n* (%)	13 (2.8)	0 (0)	13 (1.3)	7 (0.7)	6 (1.2)	13 (1.3)

Chi-square, Fisher’s Exact tests; for gender *p* < 0.05; for side *p* > 0.05

Table 3 summarizes the number of roots in relation to tooth type. Of the 500 maxillary first premolars evaluated, 199 teeth had one root (39.8%), whilst 293 (58.6%) were two-rooted. Three-rooted maxillary first premolars were found in 8 (1.6%). For maxillary second premolars, 416 premolars had one root (83.2%), whilst 79 (15.8%) had two roots and the rest 5 (1.0%) were three roots. There were significant differences of number of root were found between groups (*p* > 0.05).

**Table 3:** Number of roots in maxillary premolars

Number of roots	Gender			Tooth position		
	Male	Female	Total	Left side	Right side	Total
*First premolars*						
One root *n* (%)	68 (29.3)	131 (48.9)	199 (39.8)	105 (42.0)	94 (37.6)	199 (39.8)
Two roots *n* (%)	156 (67.2)	137 (51.1)	293 (58.6)	141 (56.4)	152 (60.8)	293 (58.6)
Three roots *n* (%)	8 (3.4)	0 (0)	8 (1.6)	4 (1.6)	4 (1.6)	8 (1.6)
*Second premolars*						
One root *n* (%)	179 (76.5)	237 (89.1)	416 (83.2)	211 (84.4)	205 (82.0)	416 (83.2)
Two roots *n* (%)	50 (21.4)	29 (10.9)	79 (15.8)	36 (14.4)	43 (17.2)	79 (15.8)
Three roots *n* (%)	5 (2.1)	0 (0)	5 (1.0)	3 (1.2)	2 (0.8)	5 (1.0)

For first premolars, Chi-square, Fisher’s Exact tests; for gender *p* > 0.05; for side *p* > 0.05

For second premolars, Chi-square, Fisher’s Exact tests; for gender *p* > 0.05; for side *p* > 0.05

**Fig. 1:**
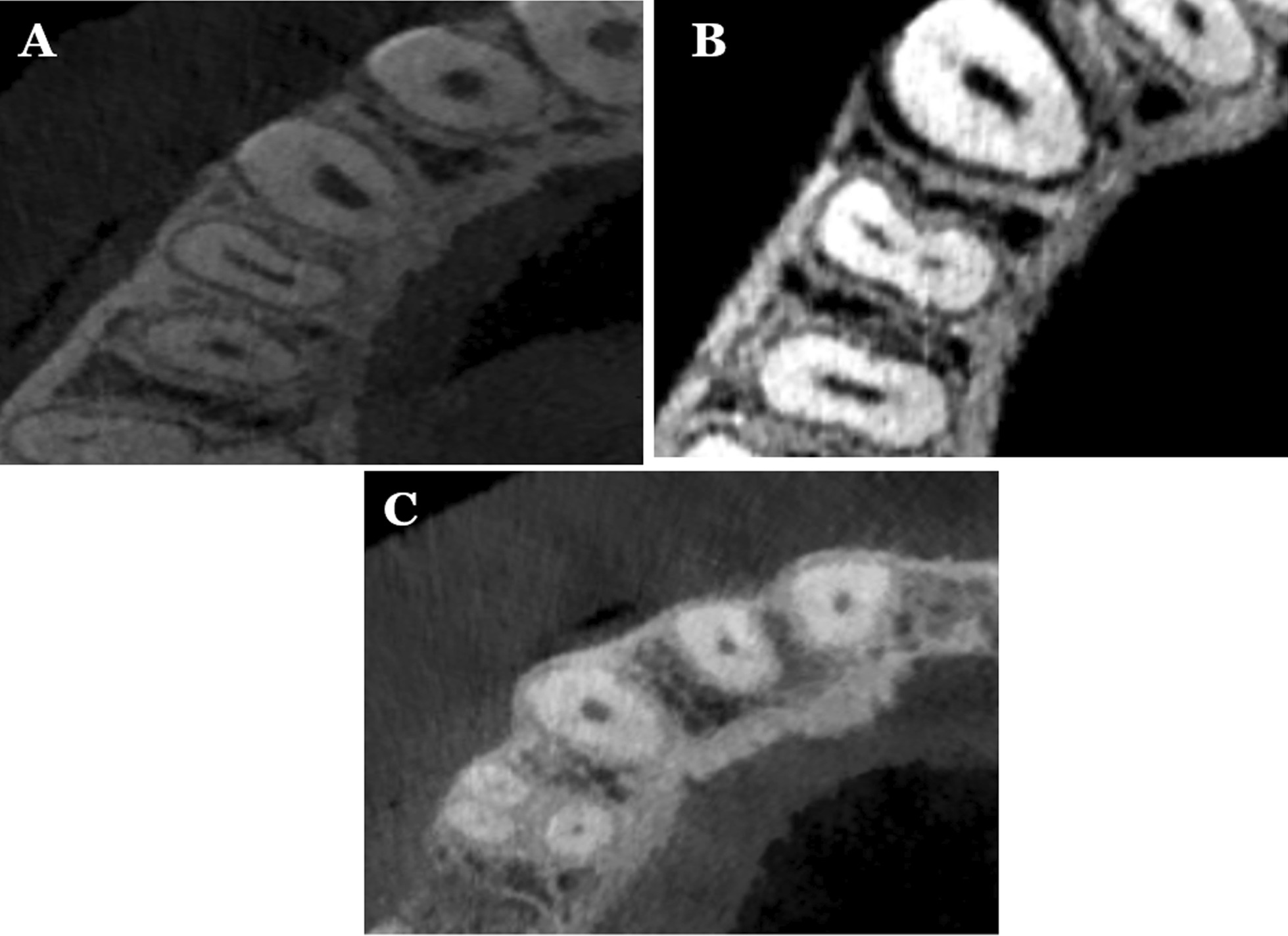
Different variations of root canal types in maxillary first premolar; **A** one canal; **B** two canals; **C** three canals

**Fig. 2:**
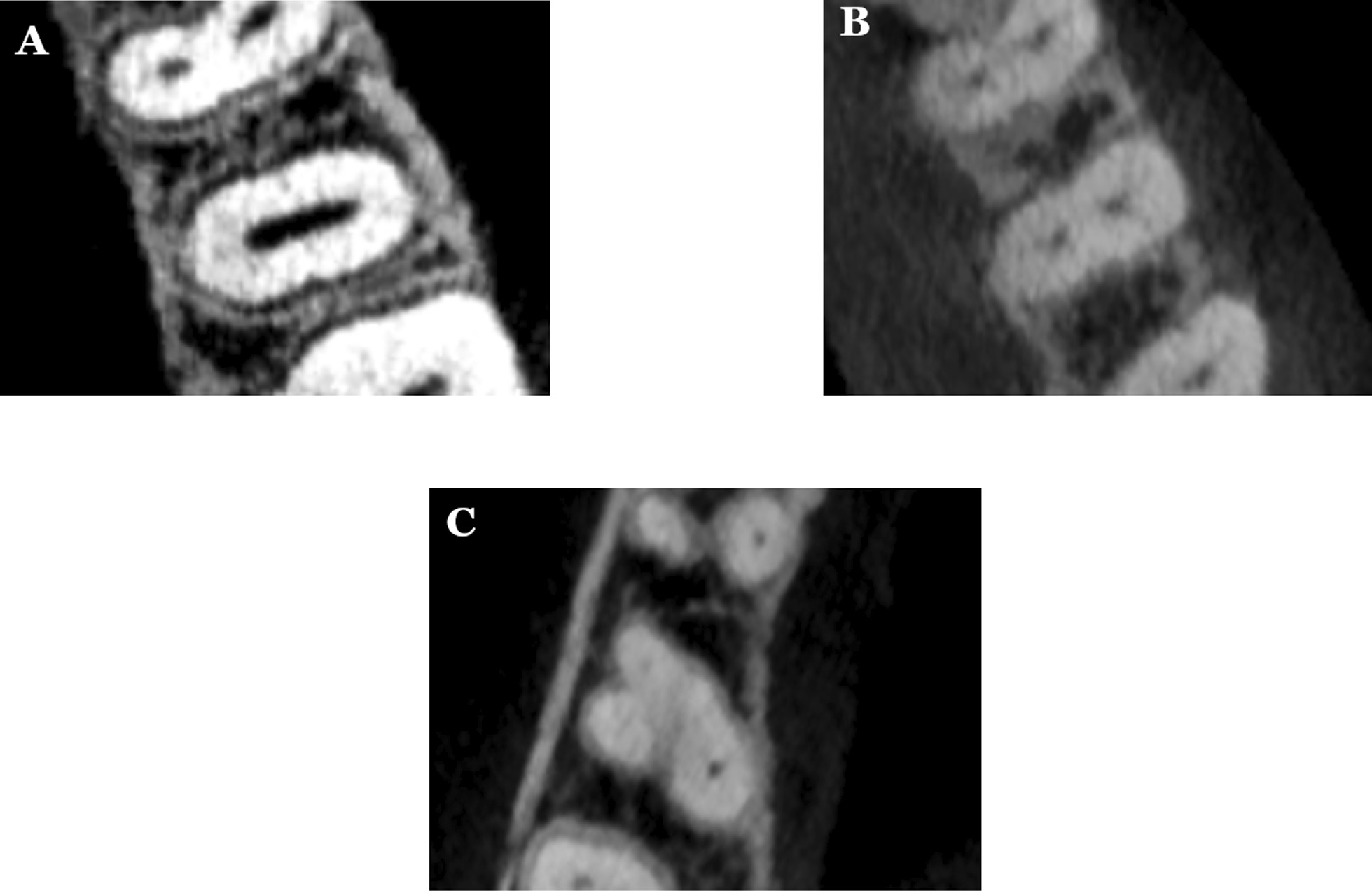
Different variations of root canal types in maxillary second premolar; **A** one canal; **B** two canals; **C** three canals

For maxillary first premolars, 39 teeth had one canal orifice (7.8%), whilst 453 (90.6%) had two canal orifices and the rest 8 (1.6%) were three root canal orifices as shown in Table 4 and Fig. 1. The three root canal orifices were totally presented in females 13 (1.3%). Of the 500 maxillary second premolars studied, 348 teeth had one canal orifice (69.6%), whilst 147 (29.4%) were two canal orifice. The three canal offices were found only in 5 samples (1.0%) orifices as shown in Table 4 and Fig. 2. A significant difference of number of root canal orifices were found between groups (*p* < 0.05).

**Table 4:** Number of canal office in maxillary premolars

Number of roots	Gender			Tooth position		
	Male	Female	Total	Left side	Right side	Total
*First premolars*						
One-orifice *n* (%)	13 (5.6)	26 (9.7)	39 (7.8)	20 (8.0)	19 (7.6)	39 (7.8)
Two-orifice *n* (%)	211 (90.9)	242 (90.3)	453 (90.6)	226 (90.4)	227 (90.8)	453 (90.6)
Three-orifice *n* (%)	8 (3.4)	0 (0)	8 (1.6)	4 (1.6)	4 (1.6)	8 (1.6)
*Second premolars*						
One-orifice *n* (%)	147 (62.8)	201 (75.6)	348 (69.6)	173 (69.2)	175 (70.0)	348 (69.6)
Two-orifice *n* (%)	82 (35.0)	65 (24.4)	147 (29.4)	74 (29.6)	73 (29.2)	147 (29.4)
Three-orifice *n* (%)	5 (2.1)	0 (0)	5 (1.0)	3 (1.2)	2 (0.8)	5 (1.0)

For first premolars, Chi-square, Fisher’s Exact tests; for gender *p* > 0.05; for side *p* > 0.05

For second premolars, Chi-square, Fisher’s Exact tests; for gender *p* > 0.05; for side *p* > 0.05

**Fig. 3:**
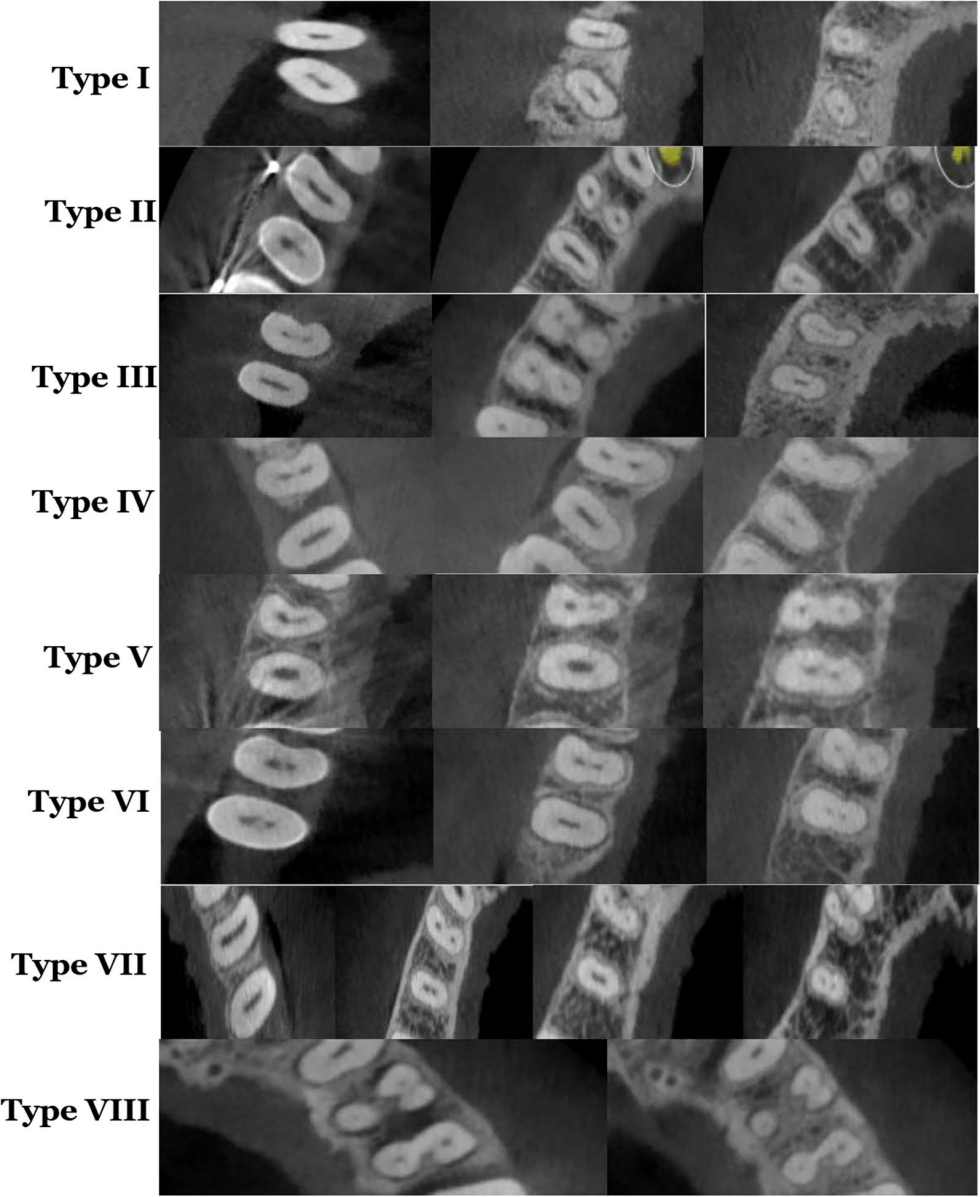
Example of Vertucci’s classification that is found in the study sample

According to Vertucci’s classification, variations in the root canal types were observed in this study shown in Fig. 3. For maxillary first premolar, Type IV was the most frequent, accounting for 57.8% of the sample (n = 289), followed by type II (32.8%, n = 164). Twenty-six of specimens (5.2%) had Vertucci type I, followed by type V in 10 (2.0%), type VIII in 8 (1.6%), while either type III were only noticed in 3 teeth (0.6%) as shown in Table 5. For maxillary second premolar, Type I was mainly occurrence 302 (60.4%), followed by Type II (16.4%, n = 82). Sixty-four of specimens (12.8%) was Type IV, followed by Types III in 32 (6.4%) and V in 14 (2.8%). The type III were noticed in five teeth (1.0%) and the type VII were only noticed in one sample (0.2%) as displayed in Table 5.

**Table 5:** Distribution of root canal types according to Vertucci’s classification

Type	I	II	III	IV	V	VII	VIII
First premolar *n* (%)	26 (5.2)	164 (32.8)	3 (0.6)	289 (57.8)	10 (2.0)	0 (0)	8 (1.6)
Second premolar *n* (%)	302 (60.4)	82 (16.4)	32 (6.4)	64 (12.8)	14 (2.8)	1 (0.2)	5 (1.0)

Chi-square, Fisher’s Exact tests; *p* < 0.05

## Discussion

For a good endodontic procedure, understanding the intricate three-dimensional root canal structure and potential diversifications is necessary. A comprehensive conceptual description; thus, an understanding of endodontic morphology can greatly reduce the difficult challenges encountered during access cavity planning, cleaning, forming, and filling procedures of the root canal system. In the literature, root canal anatomy has been identified and controversially debated [29–31].

CBCT was used in this research to examine the root structure and root canal morphologies of maxillary premolars in a Saudi subpopulation. The clinical effectiveness of endodontic procedures depends on a thorough understanding of root anatomy and the likelihood of variance in root canal pattern, as unobserved root canals can cause failure of treatment. As a result, the current research focused on the maxillary first and second premolars in order to better understand their variability in a Saudi subpopulation. The reports of the variations of premolars in the anatomic studies and the clinical cases are well mentioned in the literature and states that these are the most challenging teeth to be treated endodontically due to the wider variations in the root canal system [32]. The age, gender, ethnicity were counted as depending factors [32, 33]. For this goal, we examined a enough sample size of CBCT imaging data to decrease the sampling bias.

To accomplish an effective imagining of the root canal system, various methods [22–29] have been used. In vitro investigations have been mandated due to their dominance over in vivo investigations' inherent limitations [34]. In vivo and in vitro studies, however, can also offer useful knowledge to clinicians. In comparison to traditional 2D radiography, CBCT is an excellent tool for evaluating the root and canal morphology [28]. CBCT has been used in a number of studies to assess the morphology of maxillary premolars [35, 36]. Because of its capability to test and measure root canal anatomy in three dimensions, CBCT is said to be a better method for noticing root canal morphology than conventional periapical radiography [37, 38]. Since of the important information gained from its coronal, sagittal, and axial plans, CBCT was chosen as the assessment technique because it offers an advanced effective approach for investigating tooth exterior and internal anatomy [39]. Micro-CT, on the other hand, has a better resolution and accuracy, allowing for thorough quantitative and qualitative assessments of root canal morphology. Furthermore, micro-CT may give more anatomical details of minor anatomical characteristics such accessory canals, foramina, apical delta, and isthmi [40, 41]. However, micro-CT, is not widely available around the world. Furthermore, it is expensive and, owing to the high radiation dosages, it cannot be utilized in clinical settings [42]. Nonetheless, huge databases of previously obtained CBCT images for a number of therapeutic reasons may provide useful information on the normal root anatomy for a specific population. Furthermore, such current CBCT volumes may allow measurements and therefore quantitative study of the root and root canal diameters. Additionally, CBCT is a readily available and less expensive technique that may be employed in vivo or ex vivo [43, 44]. As a result, it was chosen for the current study to evaluate the root and root canal morphology of a maxillary premolar in a Saudi subpopulation. The data for this retrospective analysis were gathered from Ha'il city's dental clinics, which offer free dental services to a large portion of Saudi Arabia's population from various regions. A CBCT imaging database was accessed regardless of voxel size to achieve a larger sample size without exposing a large number of patients to unnecessary radiation.

The clinician can easily define and understand the degree of treatment difficulty with an appropriate root canal configuration classification. Several researchers in the literature [3, 4, 6, 45–47] categorized root canal morphology in various ways. According to Weine et al. [45], there are four kinds based on the pattern of division of a tooth's primary root canal along its length from the floor of the pulp chamber to the root apex. Meanwhile, Vertucci [46] classified root canal morphology into eight kinds, divided into three major groupings. Gulabivala et al. [3, 4] created two root canal categorization systems based on observations of root canal topologies inside mandibular molars in a sample of Burmese and Thai people. Additional kinds that were not featured in the Vertucci et al. categorization were discovered. Sert and Bayirli [6] took a new approach to root canal classification, proposing a sex-based categorization scheme for mandibular and maxillary permanent teeth in Turkish people. Fourteen new root canal designs that were not previously classified were described. Ordinola-Zapata et al. [48] evaluated C-shaped mandibular first premolars in a Brazilian subpopulation using micro-CT imaging. They discovered many novel anatomical variances and complexity in root canal anatomy that were not previously classified. Ahmed et al. [47, 49, 50] developed a novel coding system for categorizing root main and accessory canal morphology, as well as teeth with abnormalities, in order to give complete information about the tooth and its root and canal anatomical characteristics. The Vertucci classification [46] was selected as a reference in this study since it is the most commonly used classification in the literature. Despite the fact that it has been a fundamental categorization for a long time, it is still frequently employed in recent research by most authors in the literature [43, 44], and it was utilized in the current study for easy comparison with the results of other investigations. As a consequence, it was employed in this study for the reasons stated above, as well as to facilitate the comparison of results. This study, however, took into account other root canal configurations in addition to the Vertucci categorization.

The prevalence of one root was stated to be 22 to 66% in maxillary first premolars, 33 to 84% in two roots, and 0 to 6% in three roots [51–55]. The prevalence of one root was recorded to be 69.6 to 90.3% in maxillary second premolars, 9.7 to 29.7% in two roots, and 0 to 1.6% in three roots [55–58].

Atieh [60] found that the majority (80.9%) of maxillary first premolars had two roots among Saudi population, while one and three roots were found in 17.9% and 1.2%, respectively. Elkady and Allouba [59] studied the root anatomy of maxillary premolars using CBCT. They found that 28.3% of maxillary first premolars had one root and 71.7% had two roots. An important anatomical variation in maxillary premolars is the presence of three roots. This feature was reported in 0–11.7% of first premolars [59–61]. In the present study, the most commonly detected root anatomy of maxillary first was two roots (58.6%), followed by single- rooted (39.8%) and three-rooted (1.6%). The current results are in same line with Maghfuri et al. [62], who reported that the two roots were most commonly detected morphology (61%), followed by single-rooted (36%) and three-rooted (3%). In Saudi population, additional research by using CBCT were conducted. Our study were in agreement with previous reports, where two roots were 75.1%, followed by one root (23.7%) and three-rooted (1.2%) [63]. In additional report using optical radiography, sectioning methods and visual radiography in the same population, the occurrence of double-rooted in maxillary first premolars was 80.9%, followed by single-rooted 17.9%, and three-rooted 1.2% [60]. Regardless of the approach, this research provided similar findings to ours. In addition, we found a higher prevalence of two-rooted maxillary first premolar in our sample than to Yemeni (44.4%), Turkish Cypriot (44.8%), and Spanish population (51.4%), respectively [54, 58, 64]. However, we found a low incidence of single-rooted maxillary first premolars than to Yemeni populations (54.8%), North Indian populations (53.6%), and Chinese subpopulations (66%) [54, 65, 66].

In the current study, all of the specimens for maxillary first premolar corresponded to Vertucci’s classification [46]. The most common canal configuration was Type IV (57.8%), which is lower than other investigations in the same population, including Saudi Arabians (75%) [62], (69.1%) [63], and (63%) [60]. It is with the same line to other studies from Yemen (55.6%) [54], from Turkish Cypriot population (59.5%) [67]. It is also higher than in India (33.2%) [65], and in Chinese subpopulation (51%) [66].

Pecora et al. [21] reported that 90.3% of maxillary second premolars (n = 435) showed single roots, whereas 9.7% possessed two roots. Recently, Elkady and Allouba [59] found that 76.4% of maxillary second premolars found one root and 23.6% exhibited two roots. An important anatomical variation in maxillary premolars is the presence of three roots. This feature was reported in in 0–5% of second premolars [59, 61] in Saudi Arabian population. Up to three-rooted teeth were found in maxillary second premolars. Single-rooted had the highest incidence, followed by double-rooted and three-rooted (0.3%). Our finding were that 83.2% of teeth have one root, and 15.8% have two roots. Extra studies in Saudi Arabia have found one root in 76.4% and 67% of teeth, two roots in 23.6% and 30% of teeth, and three roots in 0% and 3% of teeth [59, 68].

The popular of maxillary second premolars have one root with one canal, according to popular belief [69]. Some studies maxillary second premolars had single canal between 27.70 and 48.66%, and the incidence of two canals between 50.64 and 72.30% [70]. Other researchers found a high incidence of single canals (64.1% and 67.3%) at the apex of maxillary second premolars and a comparatively low frequency of two canals (35.4% and 32.4%) in this area [9].

According to the findings, 60.4% of maxillary second premolars had only one canal. The absence or presence of three canals in maxillary second premolars has been recorded in a variety of studies, with incidences ranging from 0 to 2% of teeth [2, 71, 72]. Three canals were found in 1.0% of the total sample in this analysis, which is consistent with previous findings.

According the previous studies among a Portuguese population, woman subjects had less roots in maxillary premolars with a statistically higher in the maxillary first premolars [73]. However, in the Spanish population, there was no statistically important link between the numbers of roots and gender [35]. In the present research, there was a statistically significant connection between gender and the number of roots or gender and the root canal structure in maxillary first and second premolars, with male having more roots.

CBCT has been used to determine the symmetry in both side for root canal morphology in many studies. In Saudi patients, symmetry in right and left was found in 88.5% for the number of roots and 77% for canal pattern in maxillary first premolars [59], and symmetry of 64% was found in a Chinese population for roots number as well as root canal types [66]. Bilateral symmetry was found in 84% of maxillary second premolars for the number of roots and in 76% for canal configuration [59]. Previous studies found a high degree of symmetry in the number of roots and canal structure in maxillary second premolars, which is consistent with the current findings.

A sufficient access opening and root canal file will also aid in the discovery of extra root canals, so we recommend that in special cases, the pulp access opening be changed from the standard oval to a variety of shapes, depending on the position of the extra root canals as defined by CBCT.

The current study represented the internal root anatomy of first and second premolars in Saudi residents and, to some degree, provided a theoretical basis for clinical care. The sample size and experimental approach had a strong influence on the results of anatomical forms of root canals. There are, however, a few drawbacks that must be addressed. The sample size should have been greater because this was a single-center analysis. Furthermore, the spatial resolution of the CBCT used in this analysis was lower than that of micro- and nano-CT, which may have affected the findings. Further multicenter research using advanced techniques such as micro-CT may be able to overcome the current study's limitations.

## Conclusions

Within the limitation of the present study, it can be concluded that the race of the patient is an undeniable aspect that influences root canal anatomy. The root canal morphology of maxillary first and second premolars exposed a wide variations among Saudi subpopulations. The majority of maxillary first premolars had two roots and two root canals, while one root and one root canal was the most common anatomical configuration for maxillary second premolars. Additional canal forms do occur on occasion, and clinicians should pay close attention to them.

## Data Availability

The data that support the findings of this study are available on reasonable request from the corresponding author.

## Abbreviations

CBCT: Cone beam computed tomography
3D: Three dimension
FOV: Fields of view
DVR: Dental Volumetric Reconstruction
